# mTORc1 activity is necessary and sufficient for phosphorylation of eNOS^S1177^

**DOI:** 10.14814/phy2.13733

**Published:** 2018-06-21

**Authors:** Brandee Decker, Kevin Pumiglia

**Affiliations:** ^1^ Department of Regenerative and Cancer Cell Biology Albany Medical College Albany New York

**Keywords:** Endothelial cell, endothelial nitric oxide synthase, mammalian target of rapamycin, phosphatidylinositide 3‐kinase, rapamycin, ras homolog expressed in brain

## Abstract

Nitric oxide, produced by eNOS, plays critical roles in the regulation of vascular function and maintenance. Chronic PI3K signaling has recently been associated with vascular malformations. A well described substrate downstream of PI3K signaling is eNOS. Another critical downstream target of PI3K is the metabolic regulator, mTORc1. The relationship between mTORc1 and eNOS regulation, has not been determined. We generated cells with manipulated PI3K signaling by expressing the activating mutation, PIK3CA^H1047R^, or knocking down PTEN expression. We investigated eNOS^S1177^ phosphorylation, a major activating regulatory site, following mTORC1 inhibition. We also tested the sufficiency of mTORc1 activation to stimulate eNOS^S1177^ phosphorylation. Our data indicate mTORc1 activity is required for the phosphorylation of eNOS^S1177^, even in the presence of robust AKT activation. Moreover, we found that expression of RHEB, which functions in the absence of AKT activation to activate mTORc1, is sufficient to phosphorylate this site. Our data indicate that mTORc1, rather than AKT, may be the critical determinant of eNOS^S1177^ phosphorylation. As mTORc1 is a central regulator of cellular metabolism, the finding that this regulatory complex can directly participate in the regulation of eNOS provides new insights into metabolic uncoupling and vascular disease that often accompanies diabetes, high fat diets, and aging.

## Introduction

Nitric oxide (NO) can be generated by three isoforms of nitric oxide synthase (NOS), neuronal NOS (nNOS), inducible NOS (iNOS), and endothelial NOS (eNOS) (Forstermann and Sessa 2012). eNOS, the predominant isoform expressed within the vasculature, is responsible for most of the NO production contributing to vascular tone, angiogenesis, and vascular permeability (Nathan and Xie 1994). Its importance in regulating vascular function is underscored by the array of phenotypes exhibited in eNOS‐knockout mice, for example hypertension (Huang et al. 1995), increased vascular smooth muscle cell proliferation in response to injury (Moroi et al. 1998), increased leukocyte‐endothelial interactions (Lefer et al. 1999), hypercoagulability (Freedman et al. 1999), and exacerbated atherosclerosis within diet‐induced models (Chen et al. 2001; Kuhlencordt et al. 2001), as well as an essential role in retinal angiogenesis (Ha et al. 2016).

It is well established that cardiovascular risk factors including hypertension, obesity, and hyperglycemia are accompanied by endothelial dysfunction and often associated with changes in eNOS activity and function (Papapetropoulos et al. 1997; Cai and Harrison 2000; Fukumura et al. 2001). Interestingly, several studies have shown that these pathologies are associated with an increase, rather than decrease, in eNOS expression (Li et al. 2002). The association between enhanced eNOS expression and cardiovascular pathophysiology was confirmed in a mouse model with hyperexpression of eNOS, which when exposed to hypercholesterolemia manifests accelerated atherosclerosis (Ozaki et al. 2002). Collectively, these data support a model of a functional rheostat with respect to NO signaling and NOS expression; too little or too much can negatively impact overall cardiovascular health. Therefore, understanding the regulation of this molecule is essential to appropriately managing cardiovascular health.

eNOS has been shown to be regulated through calcium‐activated calmodulin and phosphorylation events. eNOS can be phosphorylated on several serine (Ser), threonine (Thr), and tyrosine (Tyr) residues, the most heavily studied being the phosphorylation of Serine 1177. This site is a pivotal regulator of its enzymatic activity. Phosphorylation of this site is documented to be downstream of the phosphatidylinositide 3‐kinase (PI3K) pathway, specifically Akt (Fulton et al. 1999; Huang 2009). In addition to Akt, other kinases can also phosphorylate this Serine site including AMP kinase (AMPK) (Chen et al. 1999), protein kinase A (PKA) and protein kinase G (PKG) (Butt et al. 2000), and calmodulin‐dependent kinase II (CaMKII) (Cai et al. 2008).

Epidemiologic analysis indicates that individuals with metabolic disorders have a significantly increased propensity to developing cardiovascular disease. mTORc1, a serine threonine kinase, is a well‐documented regulator of cellular metabolism (Polak and Hall 2009; Zoncu et al. 2011). Interestingly, it also serves as a central hub of signal integration of numerous protein kinases including AMPK, CAMKII, as well as AKT. Recently, vascular malformations arising from mutations in PIK3CA have been shown to be sensitive to rapamycin treatment in mice as well as humans (Limaye et al. 2015; Castel et al. 2016; di Blasio et al. 2018). Vascular malformations share several functional deficits with other vascular diseases including hypercoagulation, vascular remodeling, endothelial senescence, and inflammation. Viewing these data collectively, we hypothesized that the mTORc1 signaling axis could be a critical component and central regulator of vascular function by modulating nitric oxide signaling.

## Materials and Methods

### Cell culture

Human umbilical vein endothelial cells (HUVECS) from pooled donors were purchased from Lonza and cultured as we have previously described (Meadows et al. 2001). VEGF was added (50 ng/mL for 10 min) following a period of quiescence of 16 h in basal MCDB‐131. Rapamycin (LC Laboratories) was added to culture conditions where indicated at 0.1 ng/mL twenty‐four house prior to lysis. Experiments using doxycycline inducible vector systems cells were incubated with 1 *μ*g/mL doxycycline for at least 24 h prior to cell lysis.

### Viral infection

Low passage HUVECs were infected with a modified pSLIK lentivirus (Shinohara et al. 2007) to express PIK3CA^H1047R^ under tetracycline inducible control, as we have previously described (Bajaj et al. 2010). To silence PTEN, HUVECs were infected with the lentivirus derived from the GIPZ lentiviral vector containing mir300‐based shRNA directed against human PTEN (GE‐Dharmacon, V2LHS_92317). An activated mutant of RHEB, RHEB^S16H^ was obtained from Addgene in the pHAGE lentiviral vector (Nie et al. 2010).

### Western blotting

Western blot analysis was as previously described (Meadows et al. 2001) with the following antibodies: pAKT S473 (Cell Signaling 4060S 1:1000), AKT (Cell Signaling, 4961S 1:1000), pS6 S235/236 (Cell Signaling 4857S 1:1000), and S6 (Cell Signaling, 2217S 1:1000).

pENOS S1177 (Cell Signaling 9570S 1:1000), eNOS (Cell Signaling, 32027S 1:1000), and ERK2 (Santa Cruz Biotechnology SC‐154 1:1000). ERK2 is shown as a loading control. We have verified that expression of PIK3CA and RHEB, as well as knockdown of PTEN, does not alter the total protein expression of AKT, S6, or eNOS. All figures are representative of at least two independent HUVEC infections. Blots were imaged and quantified using a Fuji digital luminescence imager under nonsaturating conditions.

### Statistical analysis

Statistical analysis was performed using Sigma Plot. Two‐tailed Student's *t*‐tests were used for comparing two groups. For all analysis, *P* ≤ 0.05 was considered statistically significant. Bar graphs are represented as mean ± SEM.

## Results

### Phosphorylation of eNOS Serine 1177 is enhanced following PI3K activation

**Figure 1:**
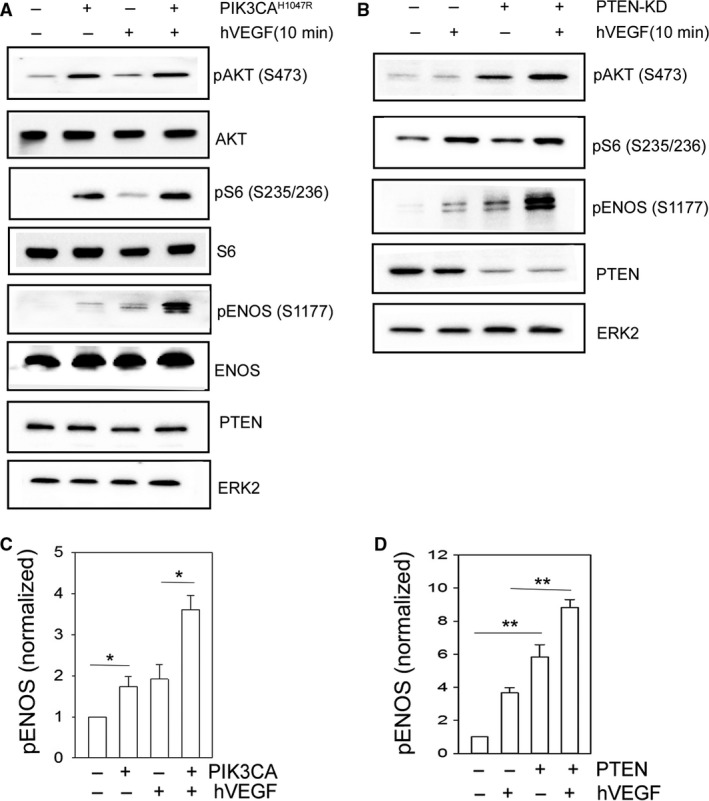
Phosphorylation of eNOS S1177 is enhanced following PI3K activation. Representative western blot analysis of pAKT (S473), pS6 (S235/236), pENOS (S1177), PTEN, and loading control ERK2 for control and PIK3CA^H1047R^ expressing HUVECs (A) and control and PTEN‐KD HUVECs (B) ± hVEGF. HUVECs were treated with hVEGF for 10 min. Total protein levels for AKT, S6, and ENOS are shown in A. Protein densitometry quantification of pENOS (S1177) for control and PIK3CA^H1047R^ expressing HUVECs (C) and control or PTEN‐KD HUVECs (D). Data are presented as mean ± SEM, *=*P* < 0.05, **=*P* < 0.001, *N* = 3. Data are representative of at least two independent infections and three western blot analyses.

PIK3CA^H1047R^ is a gain of function “hot spot” mutation within the catalytic subunit of PI3K shown to exist in cancer and vascular anomalies (Karakas et al. 2006; Gustin et al. 2008; Limaye et al. 2015; Osborn et al. 2015). We used an inducible vector to express this mutant to investigate the effects of constitutive PI3K activation on the phosphorylation of eNOS. Upon doxycycline treatment to induce expression, PIK3CA^H1047R^ transduced HUVECs show significantly enhanced phosphorylation of AKT (S473) as well as enhanced phosphorylation of S6 (S235/236), a downstream indicator of mTORc1 activity. Under these conditions, we found enhanced basal phosphorylation of eNOS (S1177), which was markedly augmented upon short‐term stimulation with human vascular endothelial growth factor (hVEGF) (Fig. 1A and C). PTEN is the negative regulator of the PI3K pathway, and loss‐of‐function mutations have likewise been reported in cancer and vascular anomalies (Tan et al. 2007; Zhu et al. 2009; Worby and Dixon 2014). Similar to results seen with the PIK3CA mutant, knockdown of PTEN (PTEN‐KD) results in enhanced phosphorylation of AKT (S473) and S6 (S235/236). The phosphorylation of eNOS (S1177) in PTEN‐KD HUVECs (Fig. 1B and D) was augmented by the combination of VEGF and PTEN knockdown.

### Phosphorylation of eNOS^S1177^ is dependent upon mTORc1 activity

**Figure 2:**
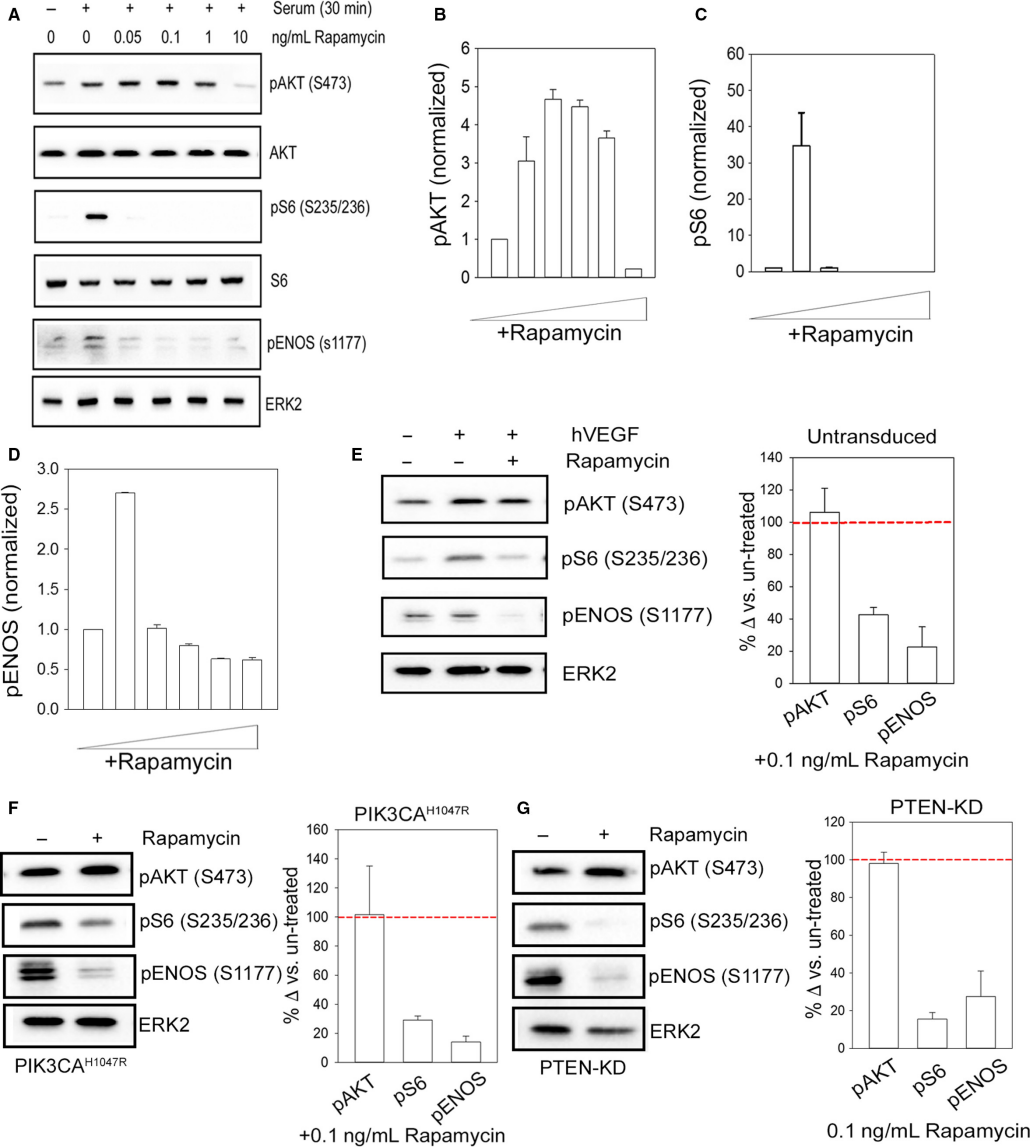
Phosphorylation of eNOS(S1177) is dependent upon mTORc1 activity. (A) Representative western blot analysis of pAKT (S473), pS6 (S235/236), and loading control ERK2 to increasing doses of rapamycin in untransduced HUVECs ± serum for 30 min. Protein densitometry quantification of pAKT (S473) (B) and pS6 (S235/236) (C) and pENOS (S1177) (D) in untransduced HUVECs. For graphical representation of pS6 (S235/236), the +Serum/−Rapamycin condition is shown at 1% of actual fold change to avoid breaking the axis. Representative western blot analysis of pAKT (S473), pS6 (S235/236), pENOS (S1177), and loading control ERK2 in untransduced HUVECs and protein densitometry quantification for pAKT (S473), pS6 (S235/236), and pENOS (S1177) for untransduced (E), PIK3CA^H1047R^ (F), and PTEN‐KD (G) HUVECs ± hVEGF ±0.1 ng/mL rapamycin. Data are presented as percent change when compared to rapamycin untreated conditions (represented by red dotted line. Data are presented as mean ± SEM. *N* = 3. Data are representative of at least two independent infections and three western blot analyses.

mTOR exists in two functionally distinct complexes mTORc1 and mTORc2. mTORc2 phosphorylates Akt on S473 (Foster and Fingar 2010). To assess whether mTORc1 signaling was involved in this phosphorylation event, we employed rapamycin, a noncompetitive inhibitor that targets mTORc1. We sought to selectively target mTORc1, without effects on the AKT/mTORc2 axis, as higher doses of rapamycin can block both mTOR complexes (as seen in Fig. 2A–C). We found doses as low as 0.1 ng/mL would reliably inhibit S6 (S235/236) and did not inhibit the phosphorylation of S473 of AKT, regulated by mTORc2 activity (Fig. 2A–C). Using rapamycin at this low dose (0.1 ng/mL), we found a marked inhibition of eNOS (S1177) phosphorylation induced by Serum containing growth media (Fig. 2A and D) as well as the angiogenic agonist, VEGF (Fig. 2D and E), reducing eNOS phosphorylation to basal levels seen with growth factor‐free conditions. Importantly, rapamycin was without effects on mTORc2‐mediated AKT phosphorylation (S473). We also examined the effect of rapamycin on the combined effect of VEGF stimulation in the background of high PI3K signaling induced by PIK3CA^H1047R^ or PTEN‐KD. Under these maximal activation conditions, rapamycin had no effect on AKT phosphorylation, which remained high, but eNOS^S1177^ phosphorylation was nearly completely inhibited (Fig. 2F, G).

### Activation of mTORc1 by RHEB expression is sufficient to phosphorylate eNOS^S1177^

**Figure 3:**
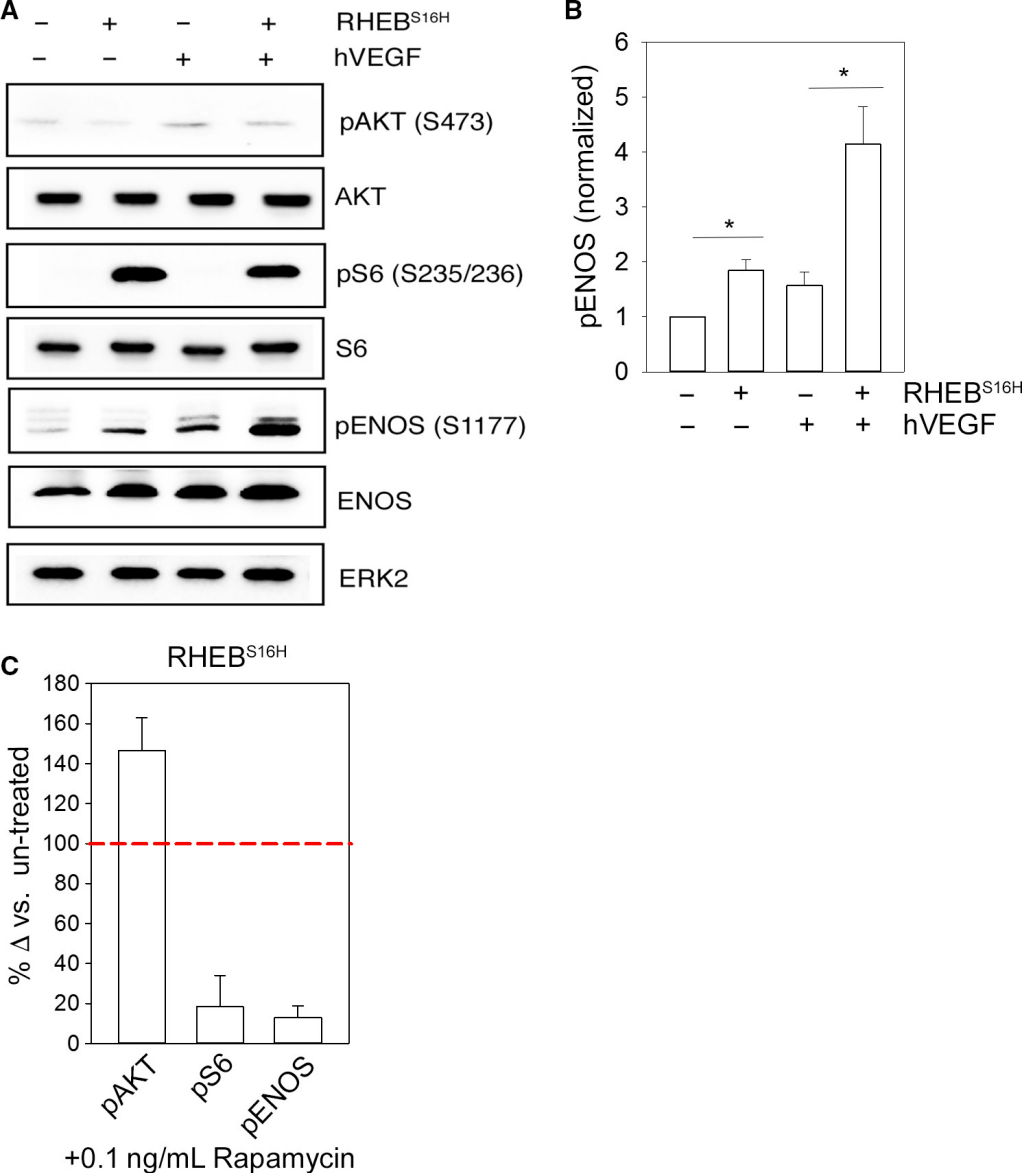
Activation of mTORc1 by RHEB expression is sufficient to phosphorylate eNOS S1177. (A) Representative western blot analysis of pAKT (S473), total AKT, pS6 (S235/235), total S6, pENOS (S1177), total eNOS and a loading control, ERK2, in control and RHEB^S16H^‐expressing HUVECs ± hVEGF. (B) Quantification of protein densitometry for pENOS (S1177) normalized to loading control ERK2 for control and RHEB^S16H^‐expressing HUVECs ± hVEGF. HUVECs were stimulated with hVEGF for 10 min. (C) Quantification of protein densitometry for pAKT (S473), pS6 (S235/236), and pENOS (S1177) for RHEB^S16H^‐expressing HUVECs +hVEGF +0.1 ng/mL rapamycin. Data are presented as percent change compared to rapamycin untreated conditions. The red dotted line is representative of untreated RHEB^S16H^ protein levels. Graphs are presented as mean ± SEM, *=*P* < 0.05 *N* = 3. Data are representative of at least two independent infections and three western blot analyses.

As mTORc1 was required for eNOS phosphorylation, we wished to determine if it was sufficient. We utilized lentiviral expression of an active RHEB^S16H^ construct in HUVECs. RHEB is a necessary and potent activator of mTORc1 (Sato et al. 2008). HUVECs transduced with active RHEB^S16H^ had significantly enhanced phosphorylation of S6 (S235/236), in the absence of any significant change in AKT activation levels. Under growth factor‐free conditions, the expression of RHEB was sufficient to induce eNOS phosphorylation at levels comparable to VEGF. The addition of hVEGF to RHEB‐expressing cells results in a marked stimulation of phosphorylation of eNOS (S1177) (Fig. 3A and B), without any corresponding augmentation of AKT phosphorylation. Treatment with low‐dose rapamycin inhibited eNOS^S1177^ phosphorylation, demonstrating that the RHEB enhancement of phosphorylation is dependent upon mTORc1 activity (Fig. 2C).

## Discussion

Our data demonstrate several important aspects of eNOS regulation including a novel mode of activation of eNOS phosphorylation at S1177. Both the pharmacological approach, with rapamycin, and genetic manipulation through mutant RHEB^S16H^ expression in HUVECs support these findings. These data underscore both the potential benefits, as well as the potential risks, of rapamycin or other mTORc1‐targeted therapies as a clinical intervention in cardiovascular disease.

Our data clearly demonstrate that eNOS phosphorylation downstream of the angiogenic agonist VEGF, as well as genetic augmentation of PI3K signaling, is not mediated directly by AKT. Rather, it seems likely that the principal role of AKT is the augmentation of mTORc1 activation, a process known to occur via modulation of TSC2 (Dibble and Cantley 2015). Furthermore, AKT activation is not required, as demonstrated by our experiments with RHEB. It is noteworthy that under conditions where mTORc1 is active, there is significant augmentation of eNOS phosphorylation by the addition of VEGF, to levels much higher than agonist stimulated levels alone. While VEGF‐stimulated eNOS phosphorylation is clearly dependent upon mTORc1, S6 phosphorylation is very robust under both PIK3CA^H1047R^ and RHEB expression conditions and is not significantly enhanced by VEGF. It will be critical to determine if the enhanced phosphorylation of eNOS^S1177^ following sustained mTORc1 activation is functionally coupled to changes in the endothelium or vasculature.

Previously VEGF stimulation has been linked to increased intracellular calcium levels and given that eNOS is regulated by calcium (Hamdollah Zadeh et al. 2008), we rationalize this may be responsible for our observed enhancement of phosphorylation. Calcium is known to enhance the translocation of eNOS to the membrane through a mechanism that is independent of phosphorylation (Pott et al. 2006). Phosphorylation of eNOS^S1177^ has been shown to stabilize the interaction between eNOS and the calcium‐binding protein, making it less dependent upon calcium (McCabe et al. 2000). It is interesting that recent data have also demonstrated that mTORc1 can also bind to and be regulated by calmodulin (Li et al. 2016). Thus, the calcium/calmodulin complex may be a critical scaffold for bringing these enzymes together and reinforcing stable interactions, leading to the high levels of phosphorylation we observed. The precise mechanistic links that exist between VEGF, calcium, and mTORc1‐dependent phosphorylation of eNOS require further investigation.

Enhanced eNOS phosphorylation, does not inevitably result in enhanced NO production and improved endothelial function. eNOS uncoupling has been documented in patients with pathological endothelial dysfunction as a result of hypercholesterolemia, diabetes mellitus, and hypertension (Forstermann and Munzel 2006). eNOS uncoupling contributes to cellular oxidative stress, the imbalance between generation and removal of free radicals, through O_2_^−^ generation. Its product NO is also sensitive to reactive oxygen species such as O_2_^−^‐forming OONO^‐^ yielding NO bioactively unavailable and ultimately further contributing to oxidative damage (Huang 2009). Importantly, phosphorylation of eNOS^S1177^ can augment superoxide generation, when uncoupled (Peng et al. 2015). Oxidative damage not only disturbs cellular metabolic function, but overall cardiac function as well. Taking our data into consideration, it is possible mTOR is the central hub of several converging signals. mTOR is a primary regulator in metabolism and as we have demonstrated here, it has the ability to modulate a critical eNOS phosphorylation site. Interestingly, mTOR is not only able to modulate oxidative stress but is also affected by it. Fully understanding the complexities and dynamics of these relationships requires a more thorough analysis in vitro and in vivo. However, in agreement with this notion, a recent study reported that enhanced S6‐kinase activity increases superoxide generation and decreases NO function, as a result of eNOS uncoupling. Rapamycin treatment was able to improve NO levels and the oxidative stress in aging rat aortas (Rajapakse et al. 2011). This previous study did not investigate eNOS phosphorylation directly; however taken together with our findings, it seems likely that the mTORc1 signaling axis phosphorylates eNOS as a consequence of S6‐kinase activation, and with persistent phosphorylation and activation drives endothelial dysfunction through uncoupling and subsequent oxidative damage.

Vascular dysfunction is a universal feature of aging and aging is an associated risk factor for cardiovascular disease. Aging has long been associated with increased mTOR activity and treatment of mice with rapamycin enhances longevity and improves survival in obese mice on a high fat diet (Wilkinson et al. 2012; Leontieva et al. 2014). Age‐associated mechanisms that contribute to endothelial dysfunction centralize around oxidative damage, defective NO signaling, increased scavenging by free radicals, and increased inflammation (Cornu et al. 2013). Given the role of mTORc1 in cellular metabolism, inflammation and now nitric oxide signaling, and the integral role these processes play in cardiovascular disease, a better understanding of the functional implications of mTORc1‐mediated phosphorylation of eNOS is required.

## Conflict of Interest

None.
